# The safety and efficacy of gabapentinoids in the management of neuropathic pain: a systematic review with meta-analysis of randomised controlled trials

**DOI:** 10.1007/s11096-022-01528-y

**Published:** 2023-02-27

**Authors:** Jawza Meaadi, Ilona Obara, Sam Eldabe, Hamde Nazar

**Affiliations:** 1 School of Pharmacy, Faculty of Medical Sciences, Newcastle University, King George VI Building, Newcastle-Upon-Tyne, NE1 7RU UK; 2 Translational and Clinical Research Institute, Newcastle University, Newcastle-Upon-Tyne, UK; 3 King Saud Medical City, Riyadh, Kingdom of Saudi Arabia; 4 Department of Pain and Anaesthesia, James Cook Hospital, Middlesbrough, UK; 5 Population Health Sciences Institute, Newcastle University, Newcastle-Upon-Tyne, UK

**Keywords:** Gabapentin, Meta-analysis, Neuralgia, Neuropathic pain, Pregabalin, Systematic review

## Abstract

**Background:**

There are increasing concerns regarding the abusive potential of gabapentinoids putting at risk patients with neuropathic pain requiring long-term pain management. The evidence to support this is rather inconcusive.

**Aim:**

This systematic review aimed to evaluate the safety and efficacy of gabapentinoids in the management of neuropathic pain with a focus on randomised controlled trials (RCTs) and categorising the side effects according to the body systems they were affecting.

**Method:**

Searches were conducted in MEDLINE (PubMed), EMBASE, Web of Science, PsycoINFO, and CINAHL (EBSCO), and included RCTs to identify and critically appraise studies investigating safety and therapeutic effects of gabapentionoids in adults with neuropathic pain. Data extraction was conducted using an established Cochrane form and the risk-of-bias tool was used in the assessment of quality.

**Results:**

50 studies (12,398 participants) were included. The majority of adverse events pertained to the nervous system (7 effects) or psychiatric (3 effects) disorders. There were more adverse effects reported with pregabalin (36 effects) than with gabapentin (22 effects). Six pregabalin studies reported euphoria as a side effect, while no studies reported euphoria with gabapentin. This was the only side effect that may correlate with addictive potential. Gabapentioids were reported to significantly reduce pain compared to placebo.

**Conclusion:**

Despite RCTs documenting the adverse events of gabapentionoids on the nervous system, there was no evidence of gabapentinoid use leading to addiction, suggesting an urgent need to design studies investigating their abusive potential.

**Supplementary Information:**

The online version contains supplementary material available at 10.1007/s11096-022-01528-y.

## Impact statements

- This systematic review and meta-analysis identified, for the first time, that the majority of adverse events with gabapentinoids were associated with their effect on the nervous system.
- Based on included RCT outcomes, there is no evidence of gabapentinoid use (maximum 20 weeks) leading to addiction, suggesting the need to design studies investigating their abusive potential.
- Critical appraisal of included RCTs indicated that gabapentinoids are effective in reducing neuropathic pain in adults.

## Introduction

The Neuropathic Pain Special Interest Group (NeuPSIG) has recommended antiepileptic drugs to manage neuropathic pain [1]. Accordingly, the United States (US) Food and Drug Administration (FDA) has permitted gabapentin treatment for postherpetic neuralgia, while pregabalin is approved for postherpetic neuralgia, neuropathic pain associated with diabetes or spinal cord injury, and fibromyalgia [2]. In the United Kingdom (UK), gabapentin and pregabalin are approved for the treatment of peripheral (both) and central (pregabalin only) neuropathic pain in adults [3, 4]. Gabapentinoids, a collective term for these drugs, have a similar structure and mechanism of action. They target α-2-δ subunit of voltage-gated calcium (Ca^2+^) channels leading to decreasing Ca^2+^ influx, subsequent neurotransmitter release (e.g., glutamate) that affects pain sensation, and results in a reduction of neuropathic pain [4, 5]. Recently, Goodman and Brett reflected that the rapid increase in prescribing of these therapeutics suggests that these are effective pain medications that are also promoted as alternatives to reduce opioid prescribing [6].

Associated with the rise in gabapentinoid use is a growing conjecture of the abuse liability. However, while there is a lack of convincing or sufficiently powerful evidence to support claims of addictive power in patients with no prior abuse history [7], it is recommended that gabapentinoid use be avoided or used in caution in patients with current or previous substance use disorders [7–9]. There has also been an increase in deaths linked to gabapentinoids which has prompted the Advisory Council on the Misuse of Drugs and the UK government to reclassify gabapentinoids as class C drugs [10–12].

### Aim

This systematic review aimed to critically appraise the evidence from randomised controlled trials (RTCs) about the safety, including addictive potential and adverse events, and analgesic efficacy of gabapentinoids to control neuropathic pain in adults. For the first time, the analysis is conducted with a focus on categorising the side effects according to the body systems and the type of the gabapentinoid administered, therefore providing a better understanding of how and which gabapentionoid affects, and potentially compromise, the therapeutic potential and safety of the medication. Our approach has been underpinned by the principles that: (1) RCTs are conventionally considered the ‘gold standard’ for evidence based medicine, (2) there is an ethical requirement to report adverse effects during RCTs, and (3) RCTs provide quantitative data that are suitable for meta-analysis to provide objective evidence.

## Method

### Search strategy

The systematic review was conducted according to the Preferred Reporting Items for Systematic Reviews and Meta-Analyses (PRISMA) guidelines [13]. Protocol methodology was registered as PROSPERO: CRD42019123869. MEDLINE (PubMed), EMBASE, Web of Science, PsycoINFO, and the Cumulative Index to Nursing and Allied Health Literature (EBSCO) were searched up to 28th June 2022. Hand searches through reference lists of key articles were also undertaken. Search terms entered into Web of Science were #1 = (“neuropathic pain” OR neuropath* OR neuralgi* OR “nerve pain”), #2 = (Gabapentin* OR Pregabalin* OR Neurontin OR Lyrica), #3 = (cancer OR neoplasm*), #4 = #1 AND #2, #5 = #4 NOT #3. The keywords used for the other databases included (pregabalin) OR (gabapentin) OR (gabapentinoids) and (neuropathic pain). The search was restricted to the English language, and there was no limitation by date.

### Study eligibility

#### Inclusion criteria

As outlined in Table 1, inclusion criteria were adopted using the PICOS [14] and focused on safety of gabapentinoids to control neuropathic pain.

**Table 1:** The PICOS elements that framed the inclusion criteria

Participants (P)	Adult patients with neuropathic pain
Intervention (I)	Gabapentinoids (pregabalin or gabapentin) to detail dose, strength, tapering procedure, concomitant medication use, length of exposure, and prior exposure to opioids
Comparison (C)	Placebos or active controls to control neuropathic pain
Outcomes (O)	*Primary outcomes:* Studies were included if they assessed the safety of gabapentinoids to control neuropathic pain *Secondary outcomes:* Analgesic effect of gabapentinoids (≥ 30 or 50% pain intensity reduction) and patient global impression of change (PGIC; much improved and very much or much improved)
Study Design (S)	Randomised controlled trials

#### Exclusion criteria

Studies that focused on animal or in-vitro studies, or paediatric patients alone were excluded.

### Types of outcome measures

#### Primary outcomes

- Participants who experienced any adverse event especially affecting the central nervous system.
- Withdrawals due to adverse events.
- Serious adverse events.
- Abuse and gabapentinoid misuse disorder.

#### Secondary outcomes

The Initiative on Methods, Measurement, and Pain Assessment in Clinical Trials (IMMPACT) definitions for moderate and substantial benefit in chronic pain studies was followed [15]. These were defined as the proportion of patients who:

- Achieved ≥ 50% pain reduction (substantial).
- Achieved ≥ 30% pain reduction (moderate).
- Reported patient global impression of clinical change (PGIC) as much or very much improved (moderate).
- Reported PGIC as very much improved (substantial).

### Study selection

All titles retrieved were reviewed by one author (JM). Two authors (JM and HN) then independently assessed the abstracts against the inclusion criteria. Papers considered as relevant were requested and assessed independently by the two authors for their suitability for inclusion and differences were resolved by discussion with a third author (IO).

### Data extraction

Data were extracted into a piloted data extraction form adapted from an established Cochrane version [16]. Two authors (JM and HN) extracted data independently and checked for agreement or discrepancies. A third author (IO) was consulted for additional review where appropriate.

### Assessment of methodological quality

The methodological quality of included studies was independently assessed by two authors (JM and HN) as recommended in the Cochrane Handbook for Systematic Reviews of Intervention [17]. The risk-of-bias tool was used for RCTs and applied by both assessors with discrepancies resolved by a third (IO).

### Statistical analysis

Meta-analysis was performed to compare the safety and efficacy of pregabalin and gabapentin *vs*. placebo. All the statistical analysis was performed using Review Manager (RevMan) [computer program; version 5.4, The Cochrane Collaboration, 2020].

Statistical heterogeneity among studies was assessed by graphically examining forest plots, and then evaluating the heterogeneity using a chi-square and I^2^ tests, with an I^2^ > 70% indicating heterogeneity [18]. The funnel plots were generated to assess the potential impact of publication bias in analyses of ≥ 10 studies [19].

The primary and secondary outcomes were pooled using the Mantel–Haenszel method within a random-effects model and presented as risk ratios (RRs) with the corresponding 95% confidence intervals (95% CIs). Number needed to harm (NNH) and number needed to treat (NNT) were calculated with the corresponding 95% CI to assess the clinical impact of the beneficial or harmful effect of the treatment. NNHs and NNTs were calculated only when the risk ratio was statistically significant.

## Results

### Literature search

**Fig. 1:**
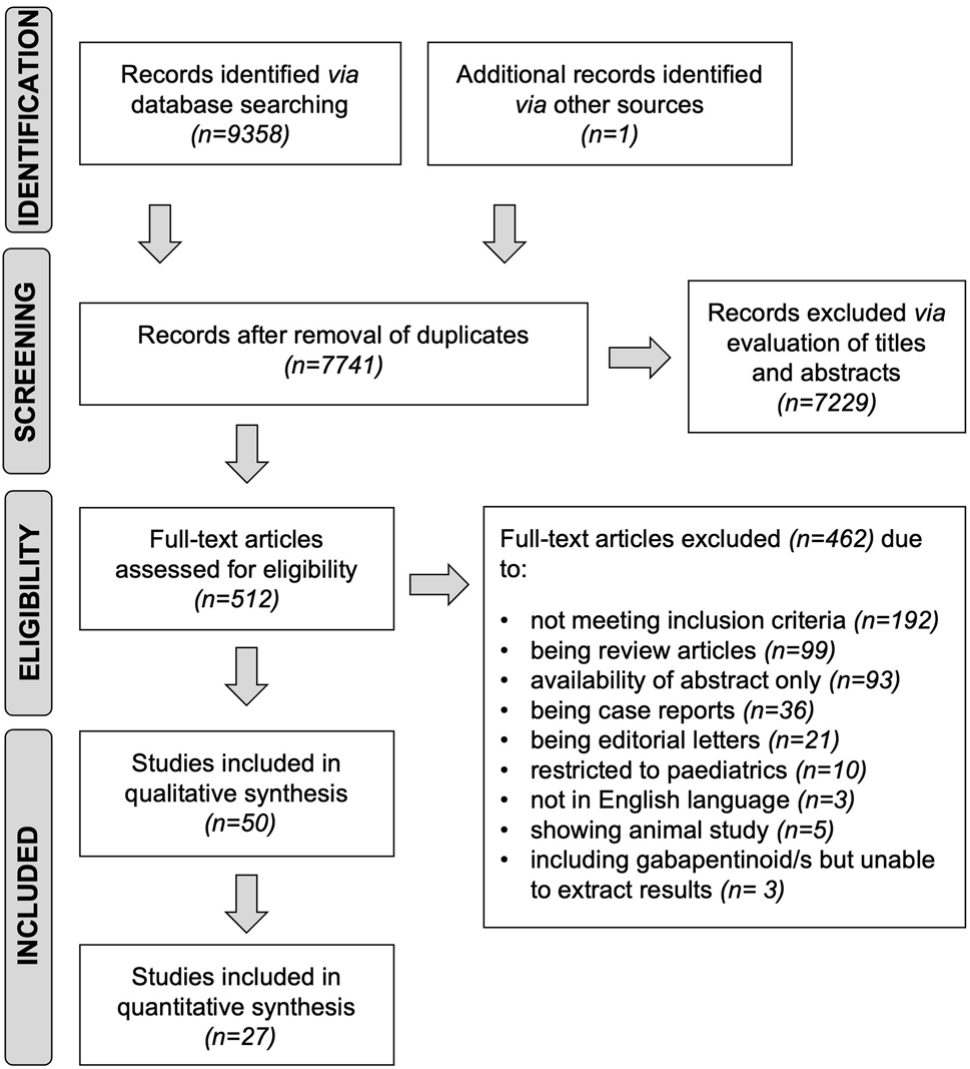
The PRISMA flow diagram detailing the search results and subsequent stages of screening

A total of 9359 titles were identified from the literature search which yielded 512 potentially relevant studies. Further assessment of the abstracts and hand searches led to 50 studies meeting the inclusion criteria (Fig. 1).

### Study characteristics

Out of the selected 50 controlled trials, 29 investigated pregabalin [2, 20–47], 16 gabapentin [48–63], and 5 studies assessed pregabalin and gabapentin compared to placebo-controlled trials [64–68]. Half of the included studies were undertaken in USA [29, 33, 35, 36, 39, 42–48, 50–52, 55, 57–60, 62–66]. Smaller numbers of studies were undertaken in India (n = 3) [30, 41, 53], China (n = 3) [2, 21, 23], UK (n = 2) [28, 56], Turkey (n = 2) [67, 68] and Japan (n = 2) [27, 31]. The review also included 1 study from Canada, Netherlands, Iran, Europe, Germany, Australia and Pakistan [20, 24, 38, 53, 61]. Nine studies were international multicentre [22, 25, 26, 32, 34, 37, 40, 49] (Supplementary material Table 1 and 2).

In total, these studies included 12,398 patients randomised to receive gabapentinoids, a placebo or a combination of drugs as comparators. Study sizes ranged from 14 to 804 participants, and the duration of the trials was 4–20 weeks.

**Fig. 2:**
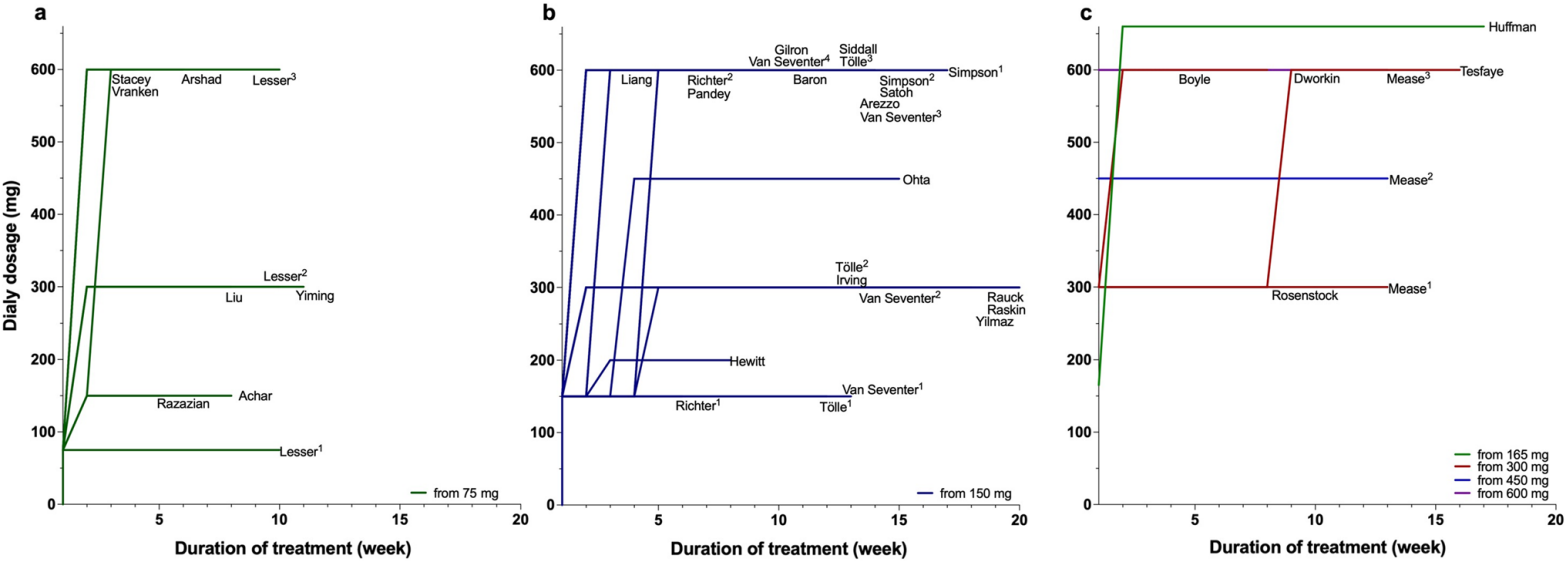
Starting dose, dose escalation and maximum daily dose achieved in selected studies for pregabalin. **a** Presents data collected for starting dose, dose escalation and duration of treatment from 75 mg. **b** Presents data collected for starting dose, dose escalation and duration of treatment from 150 mg. **c** Presents data collected for starting dose, dose escalation and duration of treatment from 165, 300, 450 or 600 mg. Superscript number 1, 2 or 3 next to the name refers to the arms in the selected study. mg; milligram

As summarised in Fig. 2, pregabalin was used at doses of 150, 300, 450 or 600 mg daily and was titrated from 75 mg daily up to the maximum dose of 300 or 600 mg daily, with titration periods between 1 and 4 weeks.

**Fig. 3:**
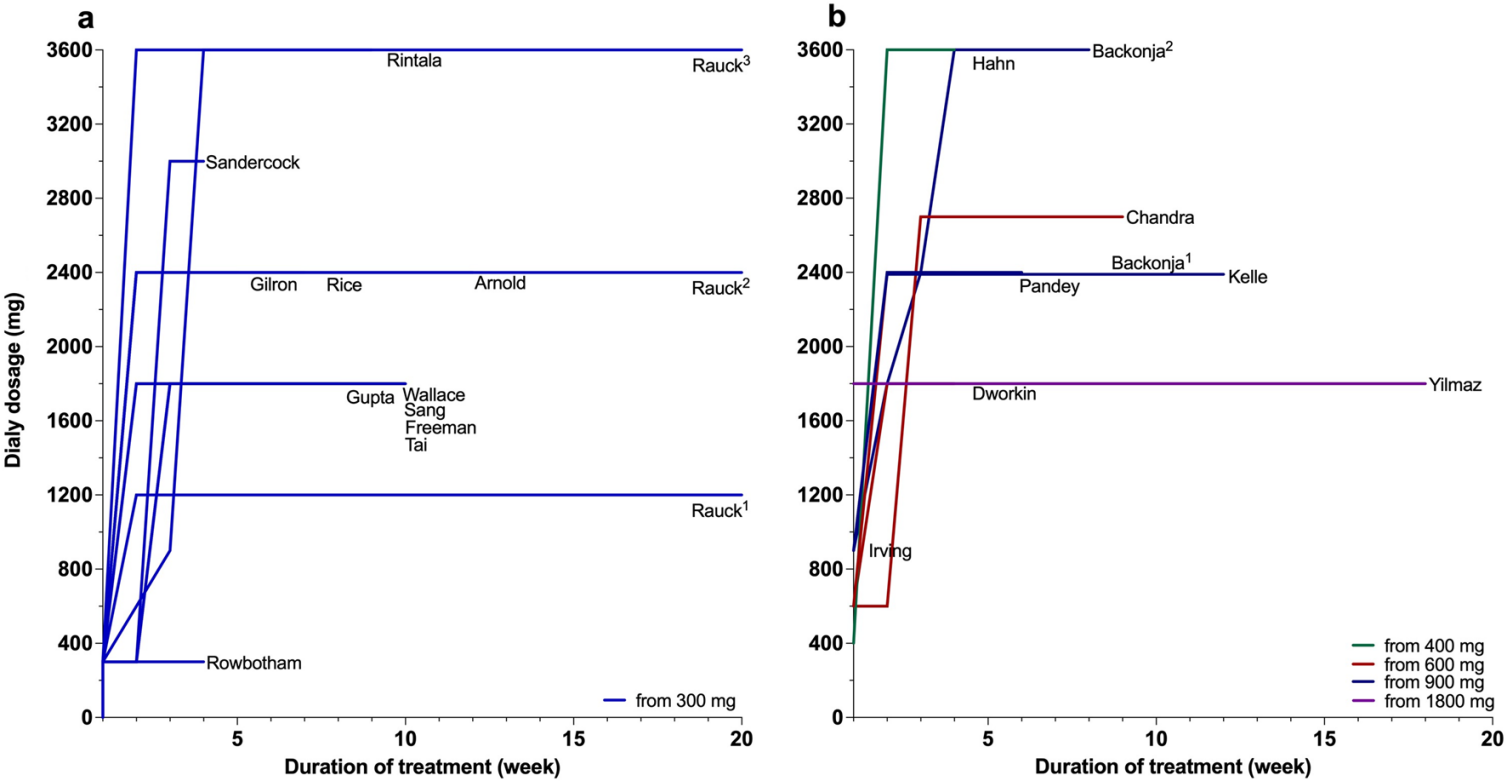
Starting dose, dose escalation and maximum daily dose achieved in selected studies for gabapentin. **a** Presents data collected for starting dose, dose escalation and duration of treatment from 300 mg. **b** Presents data collected for starting dose, dose escalation and duration of treatment from 400, 600, 900 or 1800 mg. Superscript number 1, 2 or 3 next to the name refers to the arms in the selected study. mg; milligram

As summarised in Fig. 3, gabapentin was used at doses of 1200, 1800, 2400 or 3600 mg daily, with titration periods from 1 to 8 weeks.

Additional details of included trials are shown in Supplementary material Table 1 and 2.

### Quality assessment of included studies

The quality of studies is illustrated in supplementary material Table 3. Twenty-seven studies appeared to have an unclear risk of bias, while the remaining 23 studies were considered as having a high risk of bias. These studies were excluded from the meta-analysis as has been summarised in the Supplementary material Table 4. There was no clear observable evidence of publication bias among all included studies.

### Primary outcomes (safety)

#### Reported adverse events

Most reported adverse effects pertained to a nervous system (7 effects) or psychiatric (3 effects) disorder. There were more adverse events associated with pregabalin (36 effects) than with gabapentin (22 effects) (Supplementary material Table 5). As shown in Table 2, 18 of 36 (50%) adverse events were statistically significantly associated with the pregabalin group compared to the placebo group, and 4 of 22 (18%) adverse events were significant with gabapentin treatment compared to the placebo. The highest RR (95% CI) with pregabalin treatment was found with incoordination (RR 7.21; 95% CI 1.36, 38.25), followed by abnormal gait (RR 6.71; 95% CI 1.57, 28.71), ataxia (RR 6.02: 95% CI 2.31, 31.15), euphoria (RR 6.01; 95% CI 3.02, 11.97), and increased weight (RR 4.97; 95% CI 3.08, 8.00). While gabapentin treatment had the highest RR (95% CI) with increased weight (RR 5.61; 95% CI1.04, 30.22), followed by dizziness (RR 3.33; 95% CI 2.39, 4.65), peripheral oedema (RR 3.06; 95% CI 1.25, 7.48), and somnolence (RR 2.91; 95% CI 2.10, 4.03). Analysis of adverse events data showed no evidence of heterogeneity across the studies (Supplementary material Table 5 and Figure 1).

**Table 2:** A summary of adverse events related to pregabalin and gabapentin use

Outcome	Intervention	Comparator	Studies	N	Random-effect	*P* value	I^2^ (%)	NNH (95%CI)
					RR (95%CI)			
*Nervous system disorder*								
Dizziness	Pregabalin	Placebo	27	5702	3.56 (2.91, 4.36)	< 0.00001	25	6 (4.90, 5.90)
Somnolence	Pregabalin	Placebo	18	5666	3.28 (2.62, 4.11)	< 0.00001	25	7 (6.20, 7.80)
Ataxia	Pregabalin	Placebo	5	1793	6.02 (2.31, 15.68)	0.0002	0	20 (15.6, 27.0)
Amnesia	Pregabalin	Placebo	3	652	3.38 (1.08, 10.62)	0.04	0	34 (19.3, 201.6)
Abnormal gait	Pregabalin	Placebo	3	719	6.71 (1.57, 28.71)	0.01	0	29 (18.7, 63.6)
Incoordination	Pregabalin	Placebo	3	1294	7.21 (1.36, 38.25)	0.02	0	31 (22.7, 47.4)
Asthenia	Pregabalin	Placebo	8	2544	2.00 (1.28, 3.70)	0.002	0	33 (21.3, 68.5)
*Psychiatric disorder*								
Confusion	Pregabalin	Placebo	4	1056	4.01 (1.42, 11.34)	0.002	0	30 (19.5, 62.2)
Euphoria	Pregabalin	Placebo	6	1548	6.01 (3.02, 11.97)	< 0.00001	0	16 (12.1, 22.4)
Abnormal thinking	Pregabalin	Placebo	4	1420	5.46 (2.09, 14.32)	0.0003	0	20 (14.8, 30.3)
*Eye disorder*								
Amblyopia	Pregabalin	Placebo	7	2155	2.90 (1.39, 6.03)	0.005	33	25 (17.2, 40.2)
Blurred vision	Pregabalin	Placebo	4	1306	2.59 (1.25, 5.39)	0.01	0	39 (22.5, 138.6)
*Gastro-intestinal disorder*								
Constipation	Pregabalin	Placebo	12	3838	2.49 (1.75, 3.54)	< 0.00001	0	25 (18.6, 36.0)
Dry mouth	Pregabalin	Placebo	12	3307	3.08 (2.05, 4.62)	< 0.00001	0	18 (14.0, 23.7)
*General disorder and administration site condition*								
Oedema	Pregabalin	Placebo	5	1381	2.82 (1.39, 4.74)	0.004	0	24 (16.4, 44.3)
Peripheral oedema	Pregabalin	Placebo	17	5529	2.83 (1.92, 4.17)	< 0.00001	44	22 (17.3, 29.3)
*Endocrine disorder*								
Increase weight	Pregabalin	Placebo	9	3161	4.97 (3.08, 8.00)	< 0.00001	0	16 (11.8, 17.7)
*Musculoskeletal disorder*								
Fatigue	Pregabalin	Placebo	4	838	2.00 (1.08, 3.70)	0.03	0	25 (14.0, 105.2)
*Nervous system disorder*								
Dizziness	Gabapentin	Placebo	9	2258	3.33 (2.39, 4.65)	< 0.00001	21	8 (5.90, 8.50)
Somnolence	Gabapentin	Placebo	9	2258	2.91 (2.10, 4.03)	< 0.00001	0	13 (9.50, 16.6)
*General disorder and administration site condition*								
Peripheral oedema	Gabapentin	Placebo	5	1770	3.06 (1.25, 7.48)	0.01	31	28 (19.0, 47.4)
*Endocrine disorder*								
Increase weight	Gabapentin	Placebo	2	504	5.61 (1.04, 30.22)	0.004	0	28 (16.3, 80.5)

*NNH* number needed to harm

#### Withdrawal due to adverse events

The majority of adverse events were mild to moderate in severity. The proportion of participants who withdrew due to adverse events was not reported in all the included studies. There were some studies that reported the proportion of withdrawal due to adverse events: 18 pregabalin studies [2, 21, 23, 28, 31, 37, 39, 46, 47] and 10 gabapentin studies [24, 34, 35, 45–49, 52, 61]. Adverse event withdrawals were more common with pregabalin with 314 out of 3173 participants (10%) reporting these compared to 130 out 2352 participants (6%) on placebo (RR 1.71; 95% CI 1.28, 2.29) (I^2^ = 41%; *P* = 0.0003) (NNH = 23; 95% CI 17.4, 33.6). Similarly, the proportion of participants who withdrew due to gabapentin adverse events (166/1378) (12%) were more than those participants taking the placebo (77/981) (8%) (RR 1.47; 95% CI 1.08, 2.00) (I^2^ = 21%; *P* = 0.01) (NNH = 24; 95% CI 15.1, 55.8).

#### Serious adverse events

The included studies reported that all serious adverse events were not relevant to pregabalin or gabapentin interventions and findings were not analysed.

#### Abuse and gabapentinoid misuse disorder

None of the studies assessed abuse and gabapentinoid misuse disorder.

### Secondary outcomes (efficacy)

#### Proportion of participants who achieved at least 50% pain reduction

The outcome was reported in 15 of pregabalin [21, 27, 29, 31–34, 36, 38, 39, 41–43, 46, 64] and 6 of gabapentin studies [48, 49, 51, 56, 59, 64] and the pooled results showed that pregabalin and gabapentin groups were significantly better than the placebo as presented in Table 3 (Supplementary material Figure 2).

**Table 3:** Secondary outcomes reported for pregabalin and gabapentin use

Outcome	Intervention	Comparator	Studies	N	Random-effect	*P* value	I^2^ (%)	NNT
					RR (95%CI)			
*Secondary outcomes*								
≥ 50% pain intensity reduction	Pregabalin	Placebo	15	4247	1.72 (1.37–2.16)	< 0.00001	73	10 (6.70, 10.50)
	Gabapentin	Placebo	6	1851	1.76 (1.34–2.32)	< 0.0001	54	8 (5.80, 10.80)
≥ 30% pain intensity reduction	Pregabalin	Placebo	12	3926	1.56 (1.29–1.88)	< 0.00001	79	8 (6.10, 9.80)
	Gabapentin	Placebo	7	1769	1.53 (1.25–1.88)	< 0.0001	59	7 (5.20, 9.80)
PGIC much or very much improved	Pregabalin	Placebo	13	4188	1.53 (1.28–1.83)	< 0.00001	82	9 (6.50, 10.60)
	Gabapentin	Placebo	7	1825	1.70 (1.27–2.28)	0.0004	71	7 (5.10, 9.20)
PGIC very much improved	Pregabalin	Placebo	4	1795	1.40 (1.01–1.92)	0.04	22	25 (13.8, 81.9)
	Gabapentin	Placebo	3	728	2.47 (1.79–3.41)	< 0.00001	0	6 (3.90, 7.30)

*NNT* number needed to treat, *PGIC* patient global impression of change

#### Proportion of participants who achieved at least 30% pain reduction

The proportion of participants who achieved at least a 30% pain reduction were reported in 12 of pregabalin [2, 21, 27, 29, 32, 36, 38, 42, 43, 46, 47, 64] and 7 of gabapentin studies [48, 49, 51, 52, 55, 59, 64] and the pooled results were significantly better than the placebo; but there was significant heterogeneity across the trials (Table 3).

#### Much or very much global pain improvement scale (PGIC)

The improvement in PGIC was reported in 13 of pregabalin [2, 25, 27, 29, 33, 34, 36, 39, 41, 43, 46, 47, 64] and 7 studies [48, 49, 51, 52, 56, 59, 63] comparing gabapentin against a placebo, and the pooled results indicated that pregabalin and gabapentin groups were significantly better than the placebo group but significant heterogeneity was found across the trials (Table 3).

#### Very much global pain improvement scale (PGIC)

The very much improved was reported in 4 studies with pregabalin [27, 29, 36, 47] and only 3 gabapentin studies [56, 59, 63] compared to the placebo and the pooled results demonstrated that the proportion of participants with this result was higher in pregabalin and gabapentin groups than the placebo group (Table 3).

#### Withdrawal due to lack of efficacy

Withdrawals due to lack of efficacy occurred in significantly fewer patients (3%) taking pregabalin than placebo (7%) (RR 0.41; 95% CI 0.31–0.54) (I^2^ = 4%; *P* < 0.00001) while there was no difference between those taking gabapentin compared to those on placebo (3.6%) (RR 0.59; 95% CI 0.33–1.04) (I^2^ = 0%; *P* = 0.07).

Statistical heterogeneity was noticed in some of the meta-analyses for the secondary outcomes (I^2^ ≥ 70%), this heterogeneity might be due to the included studies examining gabapentnoids with different types of neuropathic pain (i.e., postherpetic neuralgia, peripheral diabetic neuropathy, and fibromyalgia).

## Discussion

In this study, for the first time, the analysis was conducted with a focus on categorising the adverse effects according to the body systems they were affecting to better understand the safety profile associated with the use of gabapentinoinds in neuropathic pain. We identified that the majority of documented adverse events pertained to the nervous system or psychiatric disorders. Specifically, 12 of 18 (65%) adverse events were related to cognition/coordination; of these 7 pertained to a nervous system disorder (dizziness, somnolence, ataxia, amnesia, abnormal gait, incoordination, and asthenia), whereas 3 were related to a psychiatric disorder (confusion, euphoria, and abnormal thinking) and 2 to an eye disorder (amblyopia and blurred vision). This observation is in line with Perucca et al. who found that adverse events associated with the use of gabapentinoids were related to cognition/coordination and were, importantly, also the main issues impairing health-related quality of life for patients who used these medications [69]. In addition, Zaccara et al. reported that the adverse events with the highest RRs in the use of pregabalin were related to cognition/coordination [70]. This also corroborates our findings for pregabalin with the highest RRs between 3.33 and 7.20 for cognition/coordination adverse events.

Based on the included RCT outcomes, we did not detect clear indication about the abusive potential of gabapentinoids. One of the reported adverse effects that may suggest abusive potential could be euphoria resulting from the treatment with this medication. While we found 6 of 29 pregabalin studies reporting euphoria as an adverse event, no gabapentin studies reported euphoria as an adverse event. In addition, in a recently published systematic review about the abuse potential of pregabalin from 102 RCTs, euphoria was reported in 14 RCTs as an adverse event with rates between 1–10%, but 1 study reported a rate as high as 26% [71]. The reason behind the ability for pregabalin to produce euphoria, in contrast to gabapentin, may lay in the fact that the peak plasma concentration for pregabalin is achieved after 1 h of oral administration, whereas it takes between 4 and 5 h for gabapentin to reach the peak plasma concentration. This may suggest that pregabalin has rapid absorption and very high bioavailability compared to gabapentin (> 90% for pregabalin vs. 33–66% for gabapentin) [72] hence pregabalin may have higher abuse liability than gabapentin.

Even though our study design did not focus on opioid and gabapentinoid drug combination, it should be noted that gabapentinoid misuse is significantly higher in patients taking the drug in combination with an opioid analgesic where that opioid is being misused [54, 73]. Indeed, gabapentinoids have GABA-mimetic properties that may lead to drug dependence, especially in patients with a history of opioid abuse [8, 28, 54] and patients, showing long-term opioid tolerance, may desire the euphoric effect resulting from treatment with pregabalin [75]. In line with this, it has been found that the prevalence of abuse of gabapentinoids in patients with opioid use disorders was higher in pregabalin users [8, 76, 77]. However, it seems as RCTs included in this systematic review did not allow for concomitant treatment with opioids during the study period and therefore the effect of opioid and gabapentinoid drug combination would not be possible to be assessed.

We assessed the efficacy outcomes of moderate or substantial pain relief, as defined by the IMMPACT group [15]. We found that pregabalin and gabapentin were more efficacious than placebo (≥ 30% and ≥ 50% pain intensity reduction). The NNTs of pregabalin were 8 and 10, whereas gabapentin's NNTs were 7 and 8. These findings are consistent with Finnerup et al. reporting NNT of 7.7 and 7.2 for pregabalin and gabapentin, respectively [78]. In addition, some efficacy outcomes have been reported for the PGIC much or very much improved that revealed that gabapentinoids having a superior benefit compared to placebo.

The main limitation of this study is that our outcomes are based on the analysis of data retrieved from RCTs only. While there is an ethical requirement to report adverse effects during RCTs, our outcomes suggest that RCTs may not be sufficiently powered to detect adverse effects and therefore provide solid evidence to support the safety of gabapentinoids. Moreover, included RCTs were relatively short in duration (maximum 20 weeks) and this potentially limited the possible occurence of relatively rare side effects, such as addiction and misuse disorders. In addition, subgroup analysis was not undertaken to assess the risk at different doses of gabapentinoids or in different types of neuropathic pain because the main aim was to focus on the comprehensive tolerability and safety profile of gabapentinoids.

## Conclusion

This meta-analysis presents the evidence from RCTs that confirms analgesic effectiveness of gabapentionoids in adults with neuropathic pain. However, despite RCTs documenting the adverse events of gabapentionoids on the nervous system, there was no evidence of gabapentinoid use leading to addiction and misuse disorders. The only reported side effect that may be associated with the abusive potential of gabapentionoids was euphoria that was observed at the therapeutic doses range for pregabalin, but not gabapentin. Given that our outcomes were limited to RCTs only, our work suggests that RCTs assessing effectiveness of gabapentionoids are not sufficiently long in duration and not sufficiently powered to detect relatively rare side effects, such as addiction and misuse disorders. Thus, there is a critical need to improve study design or new approaches to confirm the abusive potential of gabapentinoids, to better inform and educate patients and clinicians.

## Supplementary Information

Below is the link to the electronic supplementary material.

Supplementary file1 (PDF 1070 KB)
