# Supplementary material for: The safety and efficacy of gabapentinoids in the management of neuropathic pain: a systematic review with meta-analysis of randomised controlled trials
Source: Int J Clin Pharm. 2023 Feb 27;45(3):556–65. doi: 10.1007/s11096-022-01528-y (PMC10250255; doi:10.1007/s11096-022-01528-y)
Supplement: Supplementary file 1 — Supplementary file1 (PDF 1070 KB) [file 11096_2022_1528_MOESM1_ESM.pdf]

Table 1: Characteristics of studies (pregabalin).

Pregabalin											
**Author**	**Disease**	**Diagnostic test**	**Study duration (weeks)**	**Titration Phase**	**Drug regimen**	**75 mg (n)**	**150 mg (n)**	**300 mg (n)**	**450 mg (n)**	**600 mg (n)**	**Flexible dose (n)**
(Arshad et al. 2018)<br>Pakistan	Diabetic peripheral neuropathy	**10-point Likert scale:**<br>≥4 points 7 days pre-enrolment period<br>≥ 5 years history of diabetes and symptoms of pain	6	1 w	BID			160			
(Yiming MU *et al.*, 2017)<br>China	Diabetic peripheral neuropathy	**visual analog scale pain ≥50 mm**<br>≥ 1 years history of diabetes and symptoms of pain	11	1 w	BID	313					
(Liu *et al.*, 2017)<br>China	Postherptic Neuralgia	**Visual analog scale pain score ≥ 40 mm**	8	1 w	BID		111				
(Huffman *et al.*, 2017)<br>International multicentre	Postherptic Neuralgia	**Pain numeric rating scale > 4 score**<br>Pain present for >3 months after herpes zoster infection	17	4 w	OD					208	
(Pandey *et al.*, 2015a)<br>India	Diabetic peripheral neuropathy	**visual analog scale pain ≥40 mm** or **pain severity Likert scale** ≥4	6	1 w	BID					111	
(Liang *et al.*, 2015a)<br>China	Postherptic neuralgia	**Numerical rating scale ≥ 4**<br>Pain occurring within 90 days of rash onset	4	1 w	OD					150[a]	
(Razazian *et al.*, 2014a)<br>Iran	Diabetic peripheral neuropathy	**visual analogue scale ≥40 mm**<br>History of neuropathic pain for at least 3 months	5	1 w	BID		86				
(Simpson *et al.*, 2014)<br>International multicentre	Neuropathic pain with HIV-associated human immunodeficiency virus neuropathy	**Visual analog scale pain >40mm ≥ 3 months before screening**	17	4 w	OD					183[a]	
(Raskin *et al.*, 2014)<br>International multicentre	Diabetic peripheral neuropathy	**visual analog scale pain** ≥40 mm or **pain severity Likert scale** ≥4<br>≥ 3 months dignosis of painful diabetic distal symmetrical sensorimotor polyneuropathy	20	4 w	OD			147[b]			
(Irving *et al.*, 2014)<br>USA	Diabetic peripheral neuropathy	**Pain numeric rating scale > 4**	12	1 w	BID or TID			138			
(Ohta *et al.*, 2012)<br>Japan	Fibromyalgia	**Pain numeric rating scale > 4** or **visual analog scale pain ≥40 mm**<br>Fibromyalgia Diagnostic Criteria*	15	3 w	BID				451		
(Boyle *et al.*, 2012)<br>UK	Diabetic peripheral neuropathy	**Leeds Assessment of Neuropathic Symptoms and Signs score > 12**<br>≥ 1 years history of neuropathic pain of diabetes	4	1 w	BID					27	

Pregabalin

Author	Disease	Diagnostic test	Study duration (weeks)	Titration Phase	Drug regimen	75 mg (n)	150 mg (n)	300 mg (n)	450 mg (n)	600 mg (n)	Flexible dose (n)
(Simpson *et al.*, 2010) USA	Painful HIV Neuropathy	**11-point Numerical Rating Scale ≥4** -Painful HIV-DSP for ≥ 3 months	14	2 w	OD					151	
(Achar *et al.*, 2012) India	Postherptic Neuralgia	**Visual analog scale pain ≥40 mm** Pain occurring within 30 days of rash onset	8	1 w	OD		25				
(Satoh *et al.*, 2010) Japan	Diabetic peripheral neuropathy	**11-point Numerical Rating Scale ≥4** or **visual analog scale pain ≥40 mm**	14	1 w	BID					140	
(Stacey *et al.*, 2008) International multicentre	Postherptic Neuralgia	**Visual analog scale pain ≥40 mm** -Pain present for >3 months after herpes zoster infection	4	1 w	OD			91			150-600 mg 88
(Arezzo *et al.*, 2008) USA	Diabetic peripheral neuropathy	**Visual analog scale pain ≥40 mm** Duration of painful DPN ≥3months	13	1 w	BID				82		
(Tolle *et al.*, 2008) International multicentre	Diabetic peripheral neuropathy	**Visual analog scale pain ≥40 mm** ≥ 1 years history of diabetes and symptoms of pain	12	1 w	BID		99	99		101	
(Vranken *et al.*, 2007) Netherlands	Central neuropathic pain	**Leeds Assessment of Neuropathic Symptoms and Signs score > 12** Pain persist ≥ 6 months	4	3 d	OD						150-600[d] mg 20
(Van Seventer *et al.*, 2010) International multicentre	Post-traumatic peripheral neuropathic pain	**Visual analog scale pain ≥40 mm** Post-traumatic peripheral neuropathic pain, confirmed by a pain specialist, Pain persist ≥ 3 months	8	1 w	OD					127	
(Baron *et al.*, 2010) International multicentre	Neuropathic pain associated with chronic lumbosacral radiculopathy	**11-point Numerical Rating Scale ≥4** Pain had to be present ≥3months prior to the study, stable for ≥4 weeks	10	4 w	OD						150-600 110
(Siddall *et al.*, 2006) Australia	Central neuropathic pain associated with spinal cord injury	**Visual analog scale pain ≥40 mm or Pain numeric rating scale > 4** ≥ 1 years history symptoms of pain	12	1 w	OD					70	
(Richter *et al.*, 2005) USA	Diabetic peripheral neuropathy	**Visual analog scale pain ≥40 mm or Pain numeric rating scale ≥ 4** 1- 5 years history of diabetes and symptoms of pain	6	2 w	OD		79			82	
(Tesfaye *et al.*, 2013) International multicentre	Diabetic peripheral neuropathy	**24-hour average pain severity of ≥4 on BPI-MSF**	8	0	OD					403	

Pregabalin

Author	Disease	Diagnostic test	Study duration (weeks)	Titration Phase	Drug regimen	75 mg (n)	150 mg (n)	300 mg (n)	450 mg (n)	600 mg (n)	Flexible dose (n)
		The diagnosis had to be confirmed by a score of ≥3 on the Michigan Neuropathy Screening Instrument at screening.									
(Rosenstock *et al.*, 2004)<br>USA	Diabetic peripheral neuropathy	**Visual analog scale pain ≥40 mm or Pain numeric rating scale ≥ 4**<br>1- 5 years history of diabetes and symptoms of pain	8	0	TID			76			
(Dworkin *et al.*, 2002)<br>USA	Postherptic Neuralgia	**-Visual analog scale pain ≥40 mm or Pain numeric rating scale ≥4 score**<br>-Pain present for >3 months after herpes zoster infection	8	1 w	OD					89	
(Lesser *et al.*, 2004)<br>USA	Diabetic peripheral neuropathy	**Visual analog scale pain ≥40 mm or Pain numeric rating scale ≥4 score**<br>Diagnosed type 1 or 2 diabetes and distal symmetric sensorimotor polyneuropathy for 1 to 5 years	10	1 w	OD	77		80		82	
(Hewitt *et al.*, 2010)<br>USA	Chronic neuropathic pain	**Pain numeric rating scale >5 , <10**<br>Pain present for >3 months after herpes zoster infection	8	2 w	OD						150-200<br>53
(Gilron *et al.*, 2011a)<br>USA	Peripheral neuropathic pain	**11-point Numerical Rating Scale ≥4**	9	1 w	OD						300-600<br>80
(van Seventer *et al.*, 2006)<br>USA	Postherptic Neuralgia	**-Visual analog scale pain ≥40 mm or Pain numeric rating scale > 4**<br>-Pain present for >3 months after herpes zoster infection	13	1 w	BID		87	98		90	
(Rauck *et al.*, 2012)<br>USA	Diabetic peripheral neuropathy	**11-point Numerical Rating Scale ≥4**<br>Diagnosed type 1 or 2 diabetes and distal symmetric sensorimotor polyneuropathy for 6 months to 5 years	20	1 w	OD			56			
(Mease *et al.*, 2008)<br>USA	Fibromyalgia	**11-point Numerical Rating Scale ≥4**	13	1BID				185	183	190	
(Yilmaz *et al.*, 2015)<br>Turkey	Neuropathic pain associated with spinal cord injury	**Leeds Assessment of Neuropathic Symptoms and Signs score > 12**	18	8 w	BID		15				
(Kelle *et al.*, 2012)<br>Turkey	Neuropathic pain due to peripheral injury	**Leeds Assessment of Neuropathic Symptoms and Signs score ≥ 12**	12	0	BID	15					

[a] up to 600 mg/day according to tolerability of side effects

[b] up to 300 mg/day according to tolerability of side effects

[c] up to 600 mg/day according to creatinine clearance

[d] increased the dose according to pain relief

OD: once daily, **BID:** twice daily, **TID:** thrice daily, **BPI-MSF**: Brief Pain Inventory Modified Short Form

* The American College of Rheumatology 1990 Criteria for the Classification of Fibromyalgia

Table 2: Characteristics of studies (gabapentin).

Gabapentin										
**Author**	**Disease**	**Diagnostic test**	**Study duration (weeks)**	**Titration Phase**	**Drug regimen**	**1200 mg (n)**	**1800 mg (n)**	**2400 mg (n)**	**3600 mg (n)**	**Flexible dose (n)**
(Wallace *et a*l.,2010)<br>USA	Postherpetic neuralgia	**Likert numerical rating scale ≥ 4**<br>Pain present for >3 months after herpes zoster infection	10	2 w	OD<br>BID		136			600 am+1200 pm<br>137
(Sang et al., 2013)<br>International multicentre	Postherpetic neuralgia	**11-point Numerical Rating Scale ≥4**<br>Persistent pain for 6 months to 5 years	10	2 w	OD		221			
(Freeman *et al.*, 2015a)<br>USA	Postherpetic neuralgia	**11-point Numerical Rating Scale ≥4**<br>Neuropathic pain for > 3 months or ≥6 months after the healing of herpes zoster skin rash	10	2 w	OD		357			
(Sandercock *et al.*, 2012)<br>Japan	Diabetic peripheral neuropathy	**pain numerical rating scale ≥ 4**<br>**-**Diagnosed type 1 or 2 diabetes and distal symmetric sensorimotor polyneuropathy for 6 months to 5 years	4	2 w	OD<br>BID				3000 mg<br>46	1800 am+1200 pm<br>50
(Backonja et al., 2011)<br>USA	Postherpetic neuralgia	**11-point Numerical Rating Scale ≥4**<br>or **visual analog scale pain ≥40 mm**<br>Diabetic neuropathy for 1-5 years	4	1 w	TID	47				
(Hahn *et al.*, 2004)<br>Germany	Painful HIV neuropathy	symptoms of painful HIV-SN, diagnosed by a neurologist based on history, as well as clinical and neurophysiological examination	4	4 D	TID			15		
(Chandra *et al.*, 2006)<br>India	Postherpetic neuralgia	**11-point Numerical Rating Scale ≥4**<br>or **visual analog scale pain ≥40 mm**<br>Pain present for >2 months after herpes zoster infection	9		TID			2700 mg<br>38		
(Arnold *et al.*, 2007)<br>USA	Fibromyalgia	**≥ 4 on BPI-MSF**<br>Fibromyalgia Diagnostic Criteria*	12	1 w	BID		75			
(Rauck *et al.*, 2012)<br>USA	Diabetic peripheral neuropathy	**11-point Numerical Rating Scale ≥4**<br>**-**Diagnosed type 1 or 2 diabetes and distal symmetric sensorimotor polyneuropathy for 6 months to 5 years	20	1 w	OD	56		56	117	
(Rice and Maton, 2001)<br>UK	Postherpetic neuralgia	**11-point Numerical Rating Scale ≥4**	7	4 D	OD		115	108		
(Gupta and Li, 2013)<br>USA	Postherpetic neuralgia	**11-point Numerical Rating Scale ≥4**	8	2 w	OD		359			

Gabapentin										
**Author**	**Disease**	**Diagnostic test**	**Study duration (weeks)**	**Titration Phase**	**Drug regimen**	**1200 mg (n)**	**1800 mg (n)**	**2400 mg (n)**	**3600 mg (n)**	**Flexible dose (n)**
		-Neuropathic pain for > 3 months or ≥6 months after the healing of herpes zoster skin rash								
(Dworkin *et al.*, 2009) USA	Acute pain in herpes zoster	Herpes zoster within 6 calendar days of rash onset The worst pain in the past 24 h ≥ 3 on numerical rating scale	4	1 w	TID		29			
(Backonja, 1998) USA	Diabetic peripheral neuropathy	**11-point Numerical Rating Scale ≥4** or **visual analog scale pain ≥40 mm** Persistent pain for 1to 5 years	8	4 w	OD				84	
(Tai *et al.*, 2002) USA	Neuropathic pain associated with spinal cord injury	**11-point Numerical Rating Scale ≥4** Neuropathic pain confirmed by an SCI physician Traumatic injury for ≥ 30 days	10	1 w	OD		7			
(Yilmaz *et al.*, 2015) Turkey	Neuropathic pain associated with spinal cord injury	**-Leeds Assessment of Neuropathic Symptoms and Signs score > 12**	18	8 w	TID		15			
(Gilron *et al.*, 2005) Canada	Diabetic peripheral neuropathy	**DPN:** distal, symmetric, sensory diabetic polyneuropathy -an unequivocal decrease in response to pin- prick, temperature, or vibration in both feet or bi- laterally decreased or absent ankle-jerk reflexes. **PHN:** Pain present for >6 months after herpes zoster infection	5	0	OD			57		
(Kelle *et al.*, 2012) Turkey	Neuropathic pain due to peripheral injury	**Leeds Assessment of Neuropathic Symptoms and Signs score ≥ 12**	12	0	OD			15		
(Rintala *et al.*, 2007) USA	Neuropathic pain associated with spinal cord injury	-**11-point Numerical Rating Scale >5** -SCI occurred at least 12 months before entering the study -Persistent pain for 6 months to 5 years	9	0	TID	38				
(Rowbotham *et al.*, 1998) USA	Postherptic neuralgia	**11-point Numerical Rating Scale ≥4** or **visual analog scale pain ≥40 mm** ≥ 3 months after healing of Herpes zoster rash skin	8	4 w	TID				113	
(Pandey *et al.*, 2015a) India	Diabetic peripheral neuropathy	**visual analog scale pain ≥40 mm** or **pain severity Likert scale** ≥4	6	1 w	BID					900-3600 mg

Gabapentin										
**Author**	**Disease**	**Diagnostic test**	**Study duration (weeks)**	**Titration Phase**	**Drug regimen**	**1200 mg (n)**	**1800 mg (n)**	**2400 mg (n)**	**3600 mg (n)**	**Flexible dose (n)**
(Irving *et al.*, 2014) USA	Diabetic peripheral neuropathy	**11-point Numerical Rating Scale ≥4**	12	1 w	OD					900 mg/day

* The American College of Rheumatology 1990 Criteria for the Classification of Fibromyalgia

OD: once daily, **BID:** twice daily, **TID:** thrice daily, **BPI-MSF**: Brief Pain Inventory Modified Short Form, **DPN:** diabetic peripheral neuropathy, **PHN:** postherptic neuralgia, **SCI:** spinal cord injury

Table 3: Quality assessment for included studies.

Study		Selection bias		Performance bias	Detection bias	Attrition bias	Reporting bias	Other bias
Author	Year	Random sequence generation	Allocation concealment	Blinding (participants and personnel)	Blinding (outcome assessment)	Incomplete outcome data	Selective reporting	Other sources of bias
Arshed	2018	Yes	Unclear	No	Unclear	No	Unclear	Unclear
MU	2018	Yes	Yes	Yes	Unclear	Unclear	Unclear	Yes
Liu	2017	Yes	Yes	Yes	Unclear	Unclear	Unclear	Unclear
Huffman	2017	Yes	Yes	Yes	Unclear	Yes	Yes	Unclear
Pandey	2015	Yes	Yes	Yes	No	Unclear	No	Unclear
Wallace	2010	Unclear	Yes	Yes	Unclear	Yes	Unclear	Unclear
Sang	2013	Yes	Unclear	Yes	Unclear	Yes	Yes	Unclear
Freeman	2015	Yes	Unclear	Unclear	Unclear	No	Yes	Unclear
Liang	2015	No	Unclear	No	Unclear	Unclear	Unclear	Unclear
Razazian	2014	Yes	Unclear	Yes	Unclear	Unclear	No	Unclear
Simpson	2014	Yes	Yes	Yes	Unclear	Unclear	Yes	Unclear
Raskin	2014	Yes	Unclear	Yes	Unclear	Unclear	Unclear	Unclear
Irving	2014	Yes	Unclear	No	Unclear	Unclear	No	Unclear
Sandercock	2012	Unclear	Unclear	Unclear	Unclear	Yes	Unclear	No
Ohta	2012	Yes	Yes	Yes	Unclear	Unclear	Yes	Unclear
Boyle	2012	Yes	Unclear	Yes	Unclear	Unclear	Unclear	Unclear
Simpson	2010	Yes	Yes	Yes	Unclear	Unclear	Yes	Unclear
Achar	2012	Unclear	No	Unclear	Unclear	Unclear	Unclear	Unclear
Satoh	2010	Yes	Unclear	Yes	Unclear	Unclear	Unclear	Unclear
Backonja	2011	Unclear	Unclear	Yes	Unclear	No	Unclear	No
Hahn	2004	Yes	Yes	Yes	Unclear	Unclear	Unclear	No
Stacey	2008	Unclear	Unclear	Yes	Unclear	Unclear	Unclear	Unclear
Arezzo	2008	Yes	Yes	Yes	Unclear	Unclear	Yes	Unclear
Tölle	2007	Unclear	Unclear	Unclear	Unclear	Unclear	Unclear	Unclear
Chandra	2006	Yes	Yes	Yes	Unclear	Unclear	Unclear	No
Vranken	2007	Yes	Yes	Yes	Unclear	Yes	Unclear	No
Arnold	2007	Unclear	Unclear	Unclear	Unclear	Yes	Unclear	Unclear
Van Seventer	2010	Yes	Yes	Yes	Unclear	Unclear	Yes	Unclear
Rauck	2013	Yes	Yes	Yes	Unclear	Unclear	Unclear	Unclear
Baron	2010	Yes	Yes	Yes	Unclear	No	Unclear	Yes
Siddall	2006	Yes	Yes	Yes	Unclear	Unclear	Yes	Unclear
Richter	2005	Yes	Unclear	Yes	Unclear	Unclear	Yes	Unclear
Tesfaye	2013	Yes	Yes	Yes	Unclear	Yes	Unclear	Unclear
Rosenstock	2004	Yes	Yes	Unclear	Unclear	Unclear	Unclear	Unclear
Rice	2001	Yes	Yes	Yes	Unclear	Unclear	Unclear	Unclear
Dworkin	2003	Yes	Yes	Yes	Unclear	Yes	Unclear	Unclear
Lesser	2004	Yes	Yes	Yes	Unclear	Unclear	Unclear	Unclear
Gupta and Li	2013	Unclear	Unclear	Yes	Unclear	No	Unclear	Unclear
Hewitt	2010	Unclear	Unclear	Unclear	Unclear	No	Unclear	Unclear
Gilron	2011	Yes	Yes	Yes	Unclear	Unclear	No	No
Dworkin	2009	Yes	Unclear	No	Unclear	Unclear	Unclear	No
Van Seventer	2006	Unclear	Unclear	Unclear	Unclear	Unclear	Unclear	Unclear
Backonja	1998	Yes	Unclear	Yes	Unclear	No	Unclear	Unclear
Mease	2008	Unclear	Unclear	Unclear	Unclear	Unclear	Yes	Unclear
Tai	2002	Unclear	Unclear	Yes	Unclear	Unclear	Yes	No
Yilmaz	2015	Unclear	Unclear	No	Unclear	Unclear	Unclear	No
Gilron	2005	Yes	Yes	Yes	Unclear	Unclear	Unclear	No
Kelle	2012	Yes	Unclear	Unclear	Yes	Unclear	Unclear	No
Rintala	2007	Yes	Yes	Yes	Unclear	No	Unclear	No
Rowbotham	1998	Unclear	Yes	Yes	Unclear	Yes	Unclear	Unclear

Yes	No	Unclear

Table 4: Characteristics of excluded studies.

We excluded 23 studies at high risk from this meta-analysis and reasons for exclusion were:

- Non-placebo-controlled trials.
- Different study design (crossover and Enriched Enrolment with Randomised Withdrawal; EERW).
- Acute pain.
- Integrated data.
- Flexible dose.
- Did not meet the study criteria.

Study	Reason for exclusion
(Arshad and Zulfiqar, 2018)	Missing data and active comparator
(Pandey *et al.*, 2015)	Did not meet study criteria
(Freeman *et al.*, 2015b)	Integrated data from 2 included studies
(Liang *et al.*, 2015b)	Acute pain
(Razazian *et al.*, 2014b)	Active comparators
(Irving *et al.*, 2014)	Open label trial (non-randomised)
(Boyle *et al.*, 2012)	Active comparators
(Achar *et al.*, 2010)	Active comparators
(Chandra *et al.*, 2006)	Active comparators and small sample size
(Vranken *et al.*, 2008)	Active comparators and small sample size
(Baron *et al.*, 2010)	EERW
(Tesfaye *et al.*, 2013)	Flexible dose and small sample size
(Hewitt *et al.*, 2011)	EERW
(Dworkin *et al.*, 2009)	Active comparator, EERW, small sample size
(Tai *et al.*, 2002)	Crossover design, acute pain
(Yilmaz *et al.*, 2015)	Crossover design, small sample size
(Gilron *et al.*, 2005)	Crossover design, small sample size
(Kelle *et al.*, 2012)	Did not meet the study criteria, small sample size
(Rintala *et al.*, 2007)	Did not meet the study criteria, crossover
(Gupta and Li, 2013)	Integrated data from 2 included studies, did not meet the study criteria
(Huffman *et al.*, 2017)	EERW
(Raskin *et al.*, 2013)	EERW
(Gilron *et al.*, 2011b)	EERW

Table 5: Summary of identified side effects induced by pregabalin and gabapentin.

Outcome	Intervention	Comparator	Studies	N	Random-effect RR (95%CI)	*P* value	I^2 (%)	NNH
Nervous system disorder								
Dizziness	Pregabalin	Placebo	27	5702	3.56 (2.91-4.36)	<0.00001	25	6
	Gabapentin	Placebo	9	2258	3.33 (2.39-4.65)	<0.00001	21	8
Somnolence	Pregabalin	Placebo	18	5666	3.28 (2.62-4.11)	<0.00001	25	7
	Gabapentin	Placebo	9	2258	2.91 (2.10-4.03)	<0.00001	0	13
Headache	Pregabalin	Placebo	11	3726	1.00 (0.80-1.77)	0.98	0	100
	Gabapentin	Placebo	7	1769	1.24 (0.87-1.77)	0.23	0	91
Disturbance in attention	Pregabalin	Placebo	2	440	2.38 (0.91-6.25)	0.08	0	27
Nausea	Pregabalin	Placebo	6	2077	0.98 (0.55-1.77)	0.95	46	1000
	Gabapentin	Placebo	6	1294	1.22 (0.83-1.80)	0.30	0	59
Ataxia	Pregabalin	Placebo	5	1793	6.02 (2.31-15.68)	0.0002	0	20
	Gabapentin	Placebo	2	251	3.81 (0.49-29.80)	0.20	69	9
Amnesia	Pregabalin	Placebo	3	652	3.38 (1.08-10.62)	0.04	0	34
Abnormal gait	Pregabalin	Placebo	3	719	6.71 (1.57-28.71)	0.01	0	29
Incoordination	Pregabalin	Placebo	3	1294	7.21 (1.36-38.25)	0.02	0	31
Pain	Pregabalin	Placebo	3	969	0.86 (0.46-1.61)	0.64	0	112
Back pain	Pregabalin	Placebo	2	561	1.13 (0.44-2.90)	0.92	0	334
	Gabapentin	Placebo	1	354	0.85 (0.21-3.52)	083		
Asthenia	Pregabalin	Placebo	8	2544	2.00 (1.28-3.70)	0.002	0	33
	Gabapentin	Placebo	2	484	1.40 (0.64-3.09)	0.40	0	67
Psychiatric disorder								
Confusion	Pregabalin	Placebo	4	1056	4.01 (1.42-11.34)	0.002	0	30
	Gabapentin	Placebo	1	165	7.27 (0.87-60.50)	0.07	na	
Euphoria	Pregabalin	Placebo	6	1548	6.01 (3.02-11.97)	<0.00001	0	16
Abnormal thinking	Pregabalin	Placebo	4	1420	5.46 (2.09-14.32)	0.0003	0	20
Feeling abnormal	Pregabalin	Placebo	2	676	3.98 (0.78-20.42)	0.22	33	20
Eye disorder								
Amblyopia	Pregabalin	Placebo	7	2155	2.90 (1.39-6.03)	0.005	33	25
	Gabapentin	Placebo	1	150	5.00 (0.60-41.78)	0.14	na	
Blurred vision	Pregabalin	Placebo	4	1306	2.59 (1.25-5.39)	0.01	0	39
	Gabapentin	Placebo	1	354	0.51 (0.15-1.74)	0.28	na	
Diplopia	Pregabalin	Placebo	2	637	2.90 (0.36-23.44)	0.32	0	77
Ear and labyrinth disorder								
Vertigo	Pregabalin	Placebo	2	573	6.81 (0.87-53.39)	0.07	0	26
Gastro-intestinal disorder								
Diarrhoea	Pregabalin	Placebo	9	2218	1.22 (0.83-1.80)	0.31	0	1000
	Gabapentin	Placebo	4	1000	1.56 (0.88-2.77)	0.13	0	63
Vomiting	Gabapentin	Placebo	2	500	1.34 (0.42-4.32)	0.62	0	125
Constipation	Pregabalin	Placebo	12	3838	2.49 (1.75-3.54)	<0.00001	0	25
	Gabapentin	Placebo	1	354	1.41 (0.46-4.34)	0.55	na	
Flatulence	Pregabalin	Placebo	2	514	1.12 (0.29-4.31)	0.87	0	667
	Gabapentin	Placebo	1	150	1.50 (0.44-5.10)	0.52	na	
Dry mouth	Pregabalin	Placebo	12	3307	3.08 (2.05-4.62)	<0.00001	0	18
	Gabapentin	Placebo	3	383	1.55 (0.47-5.11)	0.48	45	67
General disorder and administration site condition								
Oedema	Pregabalin	Placebo	5	1381	2.82 (1.39-4.74)	0.004	0	24
	Gabapentin	Placebo	1	150	2.00 (0.79-5.05)	0.14	na	
Face oedema	Pregabalin	Placebo	2	682	2.56 (0.38-17.14)	0.33	36	48
Peripheral oedema	Pregabalin	Placebo	17	5529	2.83 (1.92-4.17)	<0.00001	44	22
	Gabapentin	Placebo	5	1770	3.06 (1.25-7.48)	0.01	31	28
Renal and urinary disorder								
Urinary tract infection	Pregabalin	Placebo	2	808	0.82 (0.34-1.99)	0.66	26	100
	Gabapentin	Placebo	1	354	1.33 (0.49-3.65)	0.58	na	

Outcome	Intervention	Comparator	Studies	N	Random-effect RR (95%CI)	*P* value	I^2 (%)	NNH
Respiratory disorder								
Nasopharyngitis	Pregabalin	Placebo	4	1279	0.95 (0.69-1.31)	0.75	0	1000
	Gabapentin	Placebo	2	806	0.79 (0.35-1.77)	0.56	0	200
Influenza	Pregabalin	Placebo	2	521	1.57 (0.80-3.10)	0.19	0	36
	Gabapentin	Placebo	1	150	0.45 (0.17-1.24)	0.13	na	
Skin and subcutaneous tissue disorder								
Hyperhidrosis	Pregabalin	Placebo	2	546	0.47 (0.03-8.01)	0.6	67	100
Accidental injury	Pregabalin	Placebo	3	730	1.15 (0.43-3.10)	0.78	26	77
Endocrine disorder								
Increase appetite	Pregabalin	Placebo	3	1112	1.93 (0.80-4.63)	0.14	0	50
	Gabapentin	Placebo	1	354	0.51 (0.13-2.01)	0.96	na	
Increase weight	Pregabalin	Placebo	9	3161	4.97 (3.08-8.00)	<0.00001	0	16
	Gabapentin	Placebo	2	504	5.61 (1.04-30.22)	0.004	0	28
Musculoskeletal disorder								
Fatigue	Pregabalin	Placebo	4	838	2.00 (1.08-3.70)	0.03	0	25
	Gabapentin	Placebo	1	354	1.88 (0.53-6.61)	0.33	na	
Other								
Infection	Pregabalin	Placebo	4	866	1.18 (0.69-2.05)	0.54	20	100

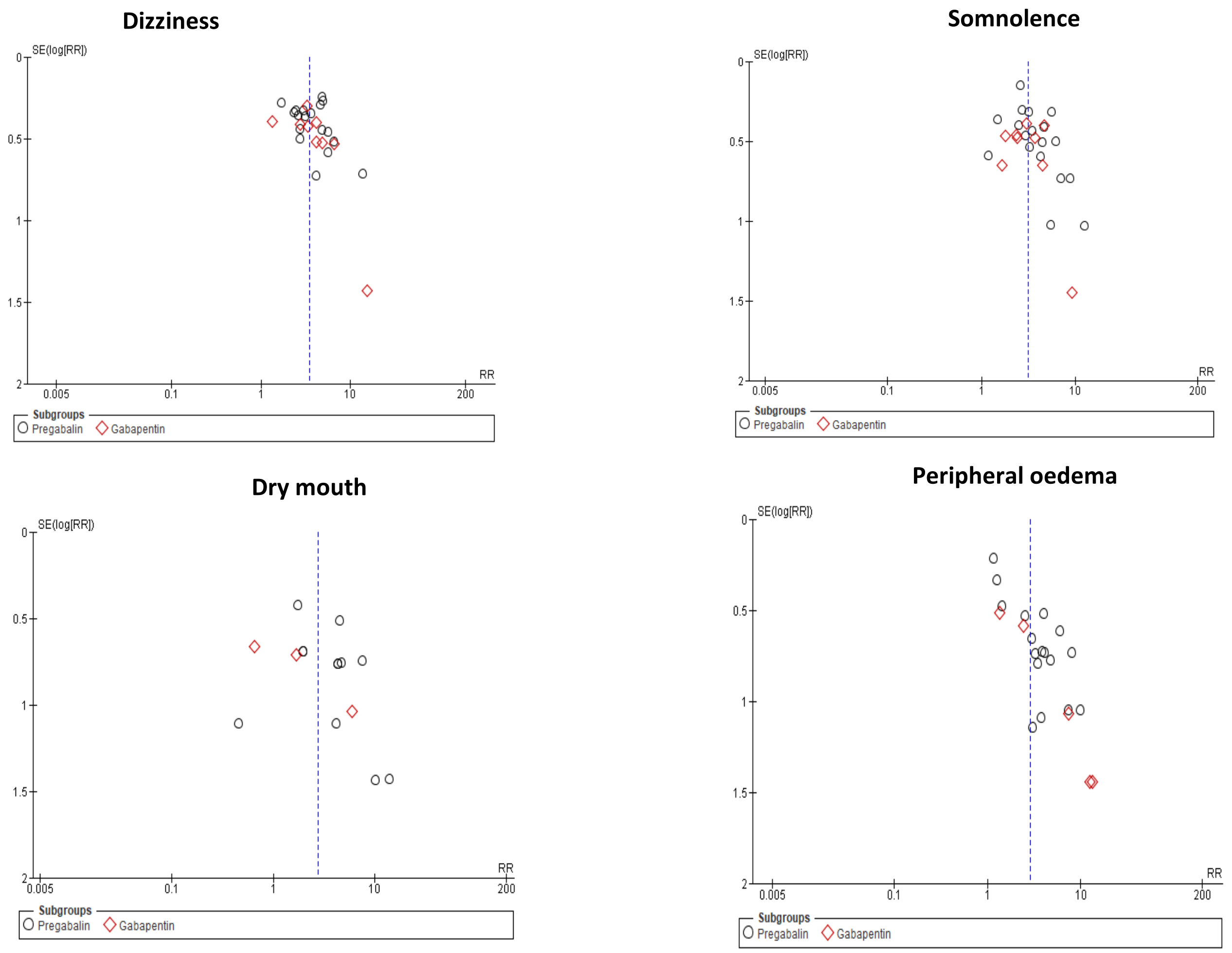

Figure 1: Funnel plot for safety and tolerability of gabapentinoids.

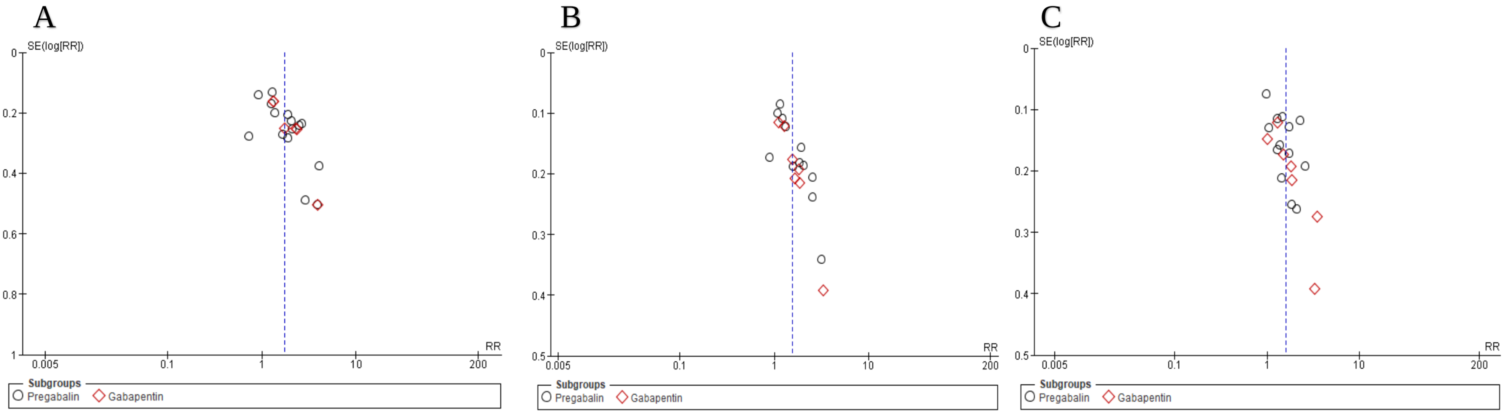

Figure 2: Funnel plots of comparison for the secondary outcomes:

A: Efficacy of gabapentinoids versus placebo: Proportion of patients with a 50% or greater reduction.

B: Efficacy of gabapentinoids versus placebo: Proportion of patients with a 30% or greater reduction.

C: Efficacy of gabapentinoid versus placebo: Proportion of patients with Improvement on PGIC scale.
